# Human Urinary mRNA as a Biomarker of Cardiovascular Disease: A Proof-of-Principle Study of Sodium Loading in Prehypertension

**DOI:** 10.1161/CIRCGEN.118.002213

**Published:** 2018-09-13

**Authors:** Brian G. Bazzell, William E. Rainey, Richard J. Auchus, Davide Zocco, Marco Bruttini, Scott L. Hummel, James Brian Byrd

**Affiliations:** 1 Departments of Internal Medicine, University of Michigan, Ann Arbor (B.G.B., R.J.A., S.L.H., J.B.B.); 2 Molecular and Integrative Physiology, University of Michigan, Ann Arbor (W.E.R.); 3 Exosomics Siena, Siena, Italy (D.Z.).; 4 Department of Life Sciences, Università degli Studi di Siena, Italy (M.B.).; 5 Section of Cardiology, Ann Arbor Veterans Affairs Medical Center, MI (S.L.H.).

**Keywords:** blood pressure, kidney, mineralocorticoids, RNA, messenger, urine

## Abstract

**Background:**

mRNA in urine supernatant (US-mRNA) might encode information about renal and cardiorenal pathophysiology, including hypertension. H, whether the US-mRNA transcriptome reflects that of renal tissues and whether changes in renal physiology are detectable using US-mRNA is unknown.

**Methods:**

We compared transcriptomes of human urinary extracellular vesicles and human renal cortex. To avoid similarities attributable to ubiquitously expressed genes, we separately analyzed ubiquitously expressed and highly kidney-enriched genes. To determine whether US-mRNA reflects changes in renal gene expression, we assayed cell-depleted urine for transcription factor activity of mineralocorticoid receptors (MR) using probe-based quantitative polymerase chain reaction. The urine was collected from prehypertensive individuals (n=18) after 4 days on low-sodium diet to stimulate MR activity and again after suppression of MR activity via sodium infusion.

**Results:**

In comparing this US-mRNA and human kidney cortex, expression of 55 highly kidney-enriched genes correlated strongly (r_s_=0.82) while 8457 ubiquitously expressed genes correlated moderately (r_s_=0.63). Standard renin-angiotensin-aldosterone system phenotyping confirmed the expected response to sodium loading. Cycle threshold values for MR-regulated targets (*SCNN1A*, *SCNN1G*, *TSC22D3*) changed after sodium loading, and MR-regulated targets (*SCNN1A, SCNN1G, SGK1*, and *TSC22D3*) correlated significantly with serum aldosterone and inversely with urinary sodium excretion.

**Conclusions:**

RNA-sequencing of urinary extracellular vesicles shows concordance with human kidney. Perturbation in human endocrine signaling (MR activation) was accompanied by changes in mRNA in urine supernatant. Our findings could be useful for individualizing pharmacological therapy in patients with disorders of mineralocorticoid signaling, such as resistant hypertension. More generally, these insights could be used to noninvasively identify putative biomarkers of disordered renal and cardiorenal physiology.

## Abstract 2

Supplemental Digital Content is available in the text.

Urine contains RNA, within and outside of cells. Renal tubular epithelial cells secrete extracellular vesicles (EVs),^1,2^ which shuttle proteins, lipids, and RNA into the urine.^3,4^ RNA-sequencing (RNA-Seq) of EVs concentrated via ultracentrifugation of supernatant from >3 L of urine has revealed a predominantly noncoding transcriptome.^5^ Beyond the noncoding transcriptome, mRNA transcripts enriched in epithelial cells of each segment of the renal tubule are detected.^4,5^ These mRNA transcripts found in urine supernatant (US-mRNA) after gentle pelleting of cells have been proposed as a noninvasive source of information about renal physiology.^4,6^ But whether US-mRNA reflects intracellular renal physiology is unknown because the transcriptional profiles of US-mRNA and human kidney have not been compared. Nor has US-mRNA been assayed in a clinical study of changing renal physiology.

A fundamental question informing the potential of US-mRNA as a biomarker of renal and cardiorenal physiology is whether the US-mRNA transcriptome reflects the transcriptome of renal tissues. To answer this question, we used publicly available RNA-Seq data to evaluate the relationship between human US-mRNA^5^ and gene expression in human renal cortex.^7^ Further proof-of-concept of the clinical usefulness of US-mRNA as a biomarker of renal and cardiorenal physiology requires detection of changes in renal physiology using US-mRNA. Thus, we also assayed US-mRNA in a clinical study of activation—and subsequent suppression—of the mineralocorticoid receptor (MR), a ligand-activated transcription factor strongly expressed in epithelial cells lining the distal renal tubule. To translate preclinical models of MR-regulated gene expression to the human setting, and with attention to clinical feasibility, we developed targeted quantitative reverse transcription polymerase chain reaction (RT-qPCR) assays of US-mRNA and applied them in small volumes of urine (5 mL). Our findings suggest that US-mRNA encodes information concordant with transcriptional activity within the kidney, supporting the feasibility of using US-mRNA to detect changes in renal gene expression.

## Methods

The data that support the findings of this study are available from the corresponding author on reasonable request. The study was submitted to and approved by the University of Michigan’s institutional review board. All participants provided written informed consent before inclusion in the study. The methods are now available as Data Supplement.

## Results

### Urinary EVs Carry Information About the Transcriptional State of Renal Tissues

**Figure 1.**
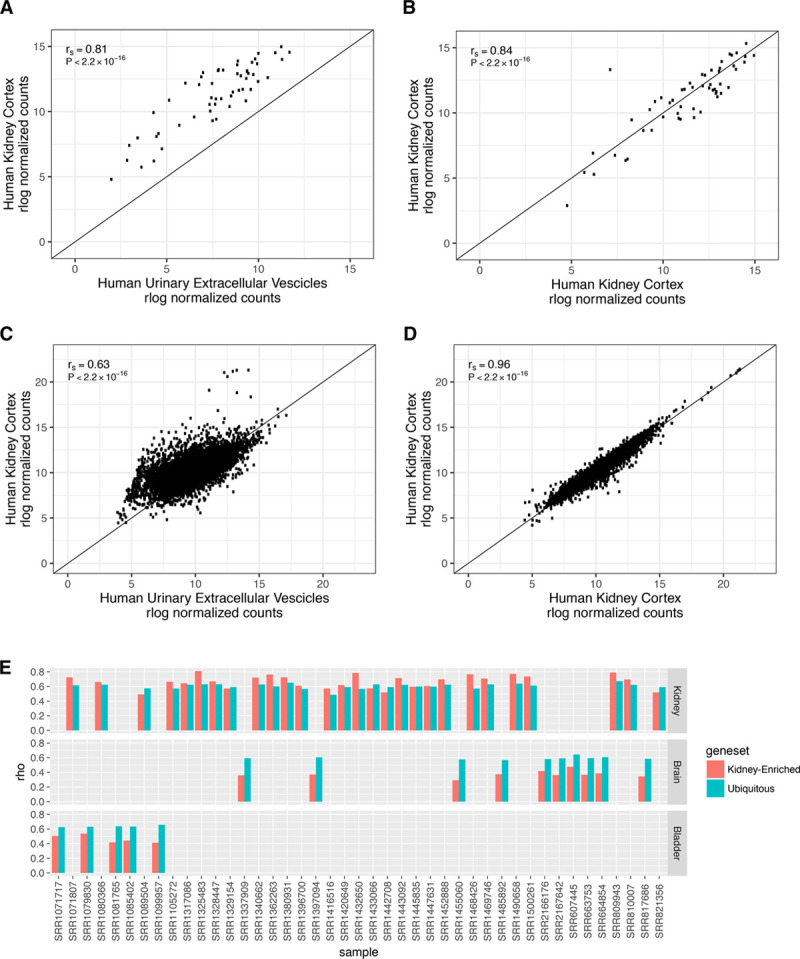
**Correlation of gene expression between transcriptomes of human urinary extracellular vesicles and a human kidney cortex, or a human kidney cortex and a second human kidney cortex sample.** **A** and **B**, Correlation for highly kidney-enriched genes; (**C** and **D**), correlation for ubiquitously expressed (housekeeping) genes. **E**, Correlation coefficients of gene expression between transcriptomes of human urinary extracellular vesicles and of a series of kidney (**top**) and brain and bladder samples (lower 2 rows, respectively, as a control). Correlations are calculated as Spearman ρ.

Whether US-mRNA has any correlation with intracellular renal gene expression is unknown. RNA in the supernatant of centrifuged urine is scant, presenting technical barriers to understanding the information encoded in US-mRNA. Overcoming several technical obstacles, Miranda et al^5^ performed RNA-Seq on EVs isolated via ultracentrifugation of 3300 mL of a healthy volunteer’s urine supernatant. We compared gene expression in this unique RNA-Seq data set to human renal cortex RNA-Seq data from the Genotype-Tissue Expression project.^7^ Samples’ data accession numbers are listed in Table I in the Data Supplement. To exclude the possibility of similarity driven by housekeeping genes, we subsequently performed separate comparisons of the RNA-Seq data sets using (1) a subset of 55 highly kidney-enriched genes (Table II in the Data Supplement) identified in Genotype-Tissue Expression, and (2) 8457 ubiquitously expressed genes identified in Genotype-Tissue Expression.^8^ For genes selected on the basis of kidney-enriched expression,^8^ we found that their relative expression in human urinary EVs correlated strongly with relative expression in a human renal cortex sample (r_s_=0.82; *P*<2.2e^−^^16^; Figure 1A). When kidney cortex samples from 2 different people were compared with each other, the strength of correlation was similar to the comparison of renal cortex and RNA from urinary vesicles (r_s_=0.84; *P*<2.2e^−^^16^; Figure 1B). In contrast, analysis of the ubiquitously expressed genes revealed moderate (r_s_=0.63; *P*<2.2e^−^^16^; Figure 1C) EV-kidney cortex correlation. As a positive control, we found a strong correlation between the expression of ubiquitous genes within the Genotype-Tissue Expression kidney cortex cohort from 2 separate people (r_s_=0.96; *P*<2.2e^−^^16^; Figure 1D). We replicated the same correlation analysis on both kidney-enriched and ubiquitous gene sets between EVs and 28 kidney cortex samples, 10 uncorrelated brain samples, and 5 uncorrelated bladder samples. Kolmogorov-Smirnov test confirmed that kidney-enriched genes correlate more strongly than ubiquitous genes in comparisons of kidney cortex versus EVs (Figure 1E); the opposite was noted in brain tissue and bladder tissue, our controls. Our findings suggest that the expression of renal genes within the human US-mRNA transcriptome is concordant with the expression of those genes within human renal tissues.

### RT-qPCR of US-mRNA Provides Proof-of-Concept for Noninvasive Detection of Changes in Human Renal Gene Transcription

In view of the preceding evidence, a logical next step is to evaluate whether changes in renal physiology could be detected in US-mRNA, ideally using a small volume of urine. The high sensitivity and specificity of locked nucleic acid probe-based RT-qPCR and the excellent state of knowledge of MR-regulated genes in animal studies facilitated our investigation of this question. The renin-angiotensin-aldosterone system regulates plasma sodium, circulating plasma volume, and blood pressure. Aldosterone regulates sodium reabsorption and water retention in the distal tubule of the nephron by binding MR which—like other nuclear hormone receptors—serves as a ligand-activated transcription factor.^9,10^ This well-characterized event leads to altered transcription of genes in distal tubule epithelial cells and,^11–15^ ultimately, sodium reabsorption.^16^ Low sodium intake results in increased renin-angiotensin-aldosterone system–mediated aldosterone signaling via MR, whereas excess sodium intake downregulates expression of a set of genes controlled by MR. With this well-established physiology in mind, we assayed cell-depleted urine samples collected under 2 conditions: (1) after 4 days on a low-sodium (20 mmol/d) diet (n=18), and (2) after subsequent sodium infusion and consumption of a high salt meal (n=17 of the same participants). An overview of the urinary RNA extraction workflow is included in Figure I in the Data Supplement, and details of the primers and probes are provided in Table III in the Data Supplement. Urinary creatinine was similar during low-sodium diet and after sodium loading (Figure II in the Data Supplement). Moreover, no clear or consistent relationship was observed between urinary creatinine and cycle threshold (C_t_) value for the RNA molecules we assayed (Figure III in the Data Supplement).

Before performing RT-qPCR analysis of RNA isolated from the 5 mL cell-depleted urine samples, we established that the study participants exhibited the expected hormonal response to sodium loading after sodium restriction. We assayed serum aldosterone concentration, plasma renin activity, urinary sodium excretion, and other relevant parameters as shown in Table IV in the Data Supplement. As expected, the renin-angiotensin-aldosterone system was more active during low-sodium diet compared with the sodium-loaded condition (*P*<0.001 for plasma renin activity and for serum aldosterone concentration; Table IV in the Data Supplement). These data are consistent with the anticipated suppression of MR signaling after sodium infusion. Consequently, urinary sodium excretion (ie, log urinary sodium-creatinine ratio) was significantly higher during the sodium-loaded condition (*P*<0.001; Figure IV in the Data Supplement). Although renin-angiotensin-aldosterone system signaling and urinary sodium excretion changed significantly from the low to high salt condition, no clinically significant change was noted in hemodynamic parameters measured by 24-hour ambulatory blood pressure monitoring.

After confirming the expected MR-mediated physiological response to sodium loading had occurred in study participants, we applied our assays for MR-regulated target genes and control genes. We assayed for 24 unique RT-qPCR targets within MR-regulated genes or genes that we anticipated would not be regulated by MR (as control genes). MR-regulated genes include *AQP2*, *SCNN1A*, *SCNN1B*, *SCNN1G*, *SGK1*, and*TSC22D3.* Control genes include *AQP1*, *HSD11B2*, *NR3C2*, and *UMOD*. A 25th assay for a RT-qPCR target within the *GAPDH* gene was performed for a subset of the participants. These assays resulted in a total of 858 sets of technical triplicates. To ensure our RT-qPCR experiment worked as expected, we performed quality control analyses. First, we determined the proportion of technical triplicates yielding a C_t_ value. C_t_ values are indirect measures of transcript abundance; lower C_t_ values correspond to higher gene expression and vice versa. We found that 645 (75.2%) of all technical triplicate sets yielded a C_t_ value for all 3 technical replicates. Few (n=17 [2.0%]) of the technical triplicate sets yielded a detectable C_t_ value for only 1 or 2 of the technical triplicates while 196 (22.8%) of the technical triplicates uniformly detected no signal (Figure V in the Data Supplement). These findings are consistent with our probe-based RT-qPCR assays having high repeatability in detecting mRNA in urine supernatant, a desirable quality for any method with prospective value in biomarker identification.

To our knowledge, no data on the distribution of C_t_ values from RT-qPCR assays of human US-mRNA molecules have been published. Using a Q-Q plot, we determined that the C_t_ values were essentially normally distributed, with few outliers (Figure VI in the Data Supplement). In addition, we found high correlation of C_t_ values within triplicate sets and high but more variable correlation of the results of different assays within a gene (Figures VII and VIII in the Data Supplement).

After we were satisfied with the technical merits of the assays, we assessed whether the detected C_t_ values were reflective of the known MR-mediated physiological response to sodium loading. The distribution of C_t_ values for MR target genes was distinct in the low and high salt conditions (Table), whereas the C_t_ values for control genes generally did not change. Four of 14 MR-regulated target RT-qPCR assays (29%) were significantly different under low sodium versus sodium-loaded condition (*SCNN1A*-2, *P*=0.01; *SCNN1G*-1, *P*=0.007; *SCNN1G*-3, *P*=0.006; *TSC22D3*-2, *P*=0.03), and a fifth assay was near statistical significance (*SGK1*-2; *P*=0.07). We evaluated whether these findings would withstand Bonferroni correction for multiple testing, which makes the conservative assumption that changes in the expression of one gene are independent of changes in the expression of another gene (which is likely not the case). The assays for *SCNN1G*-1 and *SCNN1G*-3 withstood correction for multiple testing (per Bonferroni correction for 6 MR-regulated genes, significance threshold of *P*<0.008). In contrast, only 1 of 10 RT-qPCR assays for the control genes was significantly different between the low and high salt conditions (*NR3C2*-1, *P*=0.003, likewise withstanding Bonferroni correction for 4 control genes), with the other 9 *P*>0.25.

**Table.**
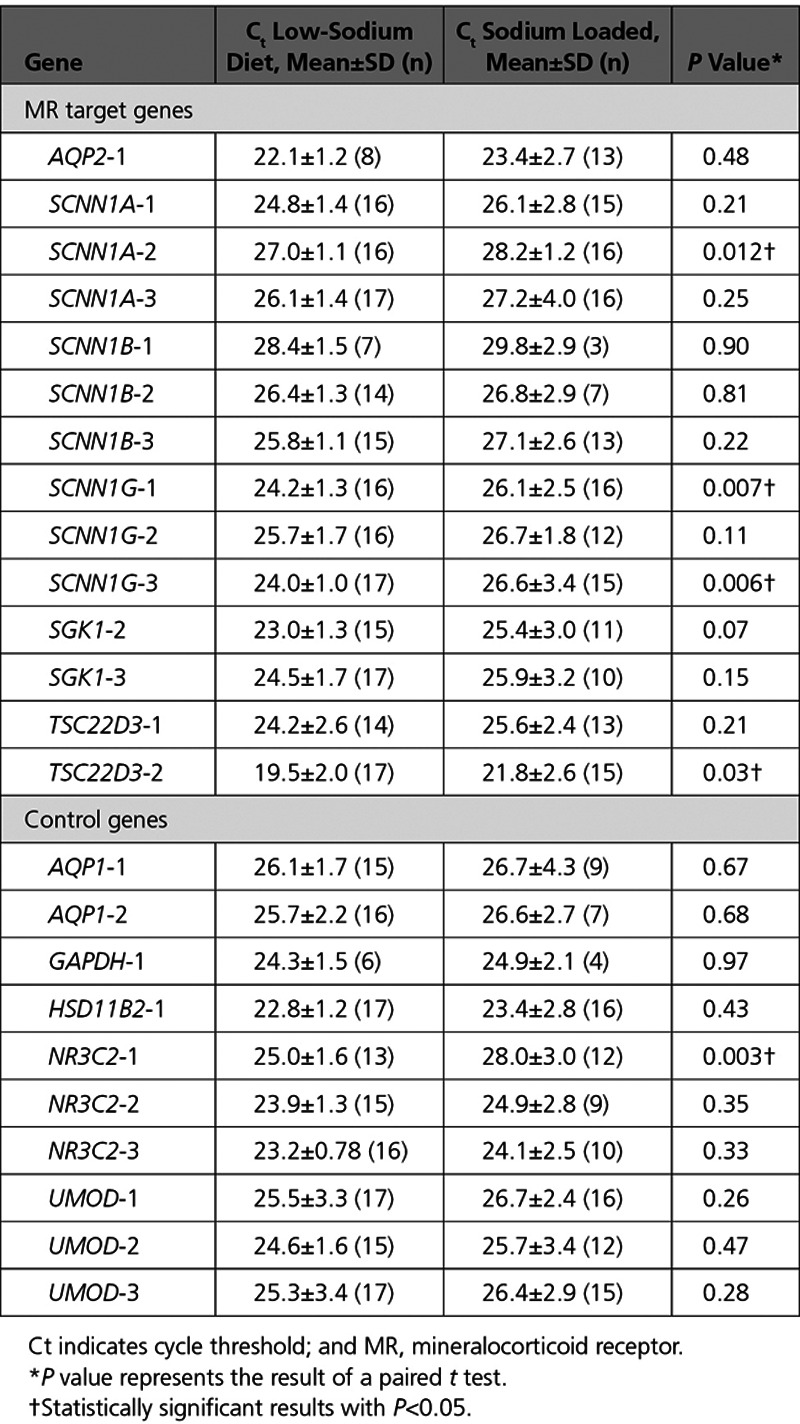
Comparison of Ct Values Under Low-Sodium Diet and Sodium-Loaded Condition

**Figure 2.**
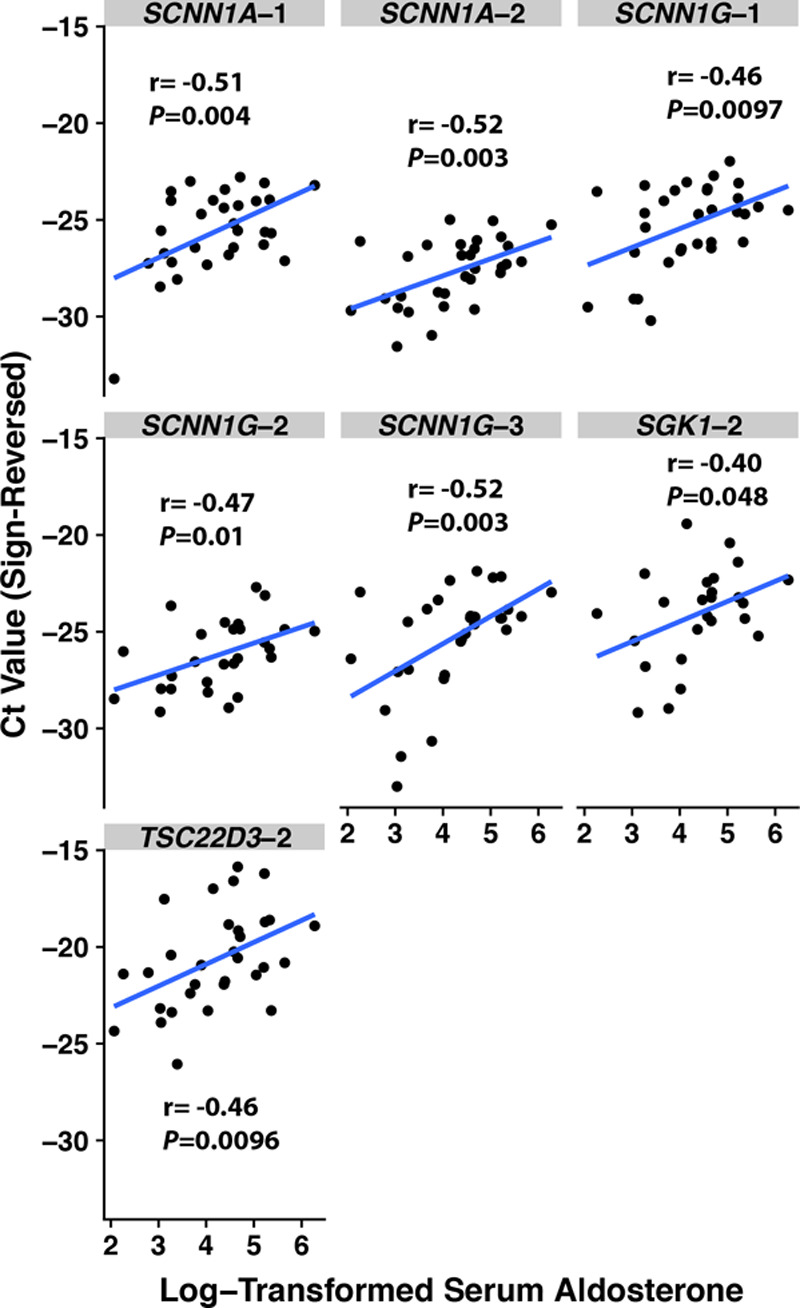
**Scatterplots of cycle threshold (C_t_) values according to log-transformed serum aldosterone, with Pearson correlation coefficients.** The C_t_ values’ sign has been reversed (from positive to negative) so that a larger C_t_ value corresponds with greater gene expression for more intuitive interpretation.

We next determined whether our results tracked well with serum levels of the canonical MR ligand, aldosterone. Higher log-transformed serum aldosterone concentration associated with significantly lower C_t_ values, and thus higher transcript abundance, for 7 of 14 (50%) of the assays for 4 target genes regulated by MR (Figure 2). *SCNN1A*-1, *SCNN1A*-2, and *SCNN1G*-3 withstood Bonferroni correction, with 3 other assays (*SCNN1G*-1, *SCNN1G*-2, *TSC22D3*-2) close to, but above, the Bonferroni correction threshold of *P*<0.008. Log-transformed serum aldosterone concentration was negatively associated with C_t_ values for 3 of 10 control gene assays, 2 of which targeted MR itself (*NR3C2*-1, *r*=−0.52, *P*=0.008; *NR3C2*-2, *r*=−0.48, *P*=0.002) and 1 which targeted uromodulin (*UMOD*-2, *r*=−0.43, *P*=0.03). Although association of several control genes with serum aldosterone might represent a chance finding, it is possible that expression of these genes is in fact altered by sodium loading.

**Figure 3.**
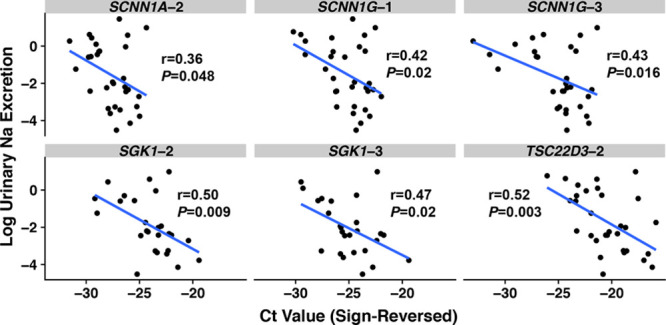
**Scatterplots of log-transformed urinary sodium-creatinine ratio according to cycle threshold (C_t_) value, with Pearson correlation coefficients.** The C_t_ values’ sign has been reversed (from positive to negative) so that a larger C_t_ value corresponds with greater gene expression for more intuitive interpretation.

We observed that 6 of 14 target gene assays, within 4 MR-regulated genes, were significantly associated with log-transformed urinary sodium-creatinine ratio (Figure 3). After Bonferroni correction, *TSC22D3*-2 withstood Bonferroni correction; *SGK1*-2 was near, but above, the correction threshold of *P*<0.008. In contrast, 0 of 10 control gene assays were significantly associated with log-transformed urinary sodium-creatinine ratio (the lowest *P* value was 0.14).

## Discussion

The major new findings of this work are (1) proof-of-concept using RNA-Seq data that the transcriptome of RNA isolated from urinary EVs is similar to the transcriptome of RNA from the kidney, (2) that human US-mRNA molecules are detectable with high fidelity in a small volume of urine, and (3) that changes concordant with known intracellular renal physiology, in this case with regard to MR activation, are detectable in US-mRNA. The ability to draw inferences about renal or cardiorenal physiology in a new, noninvasive way promises to impact the way cardiovascular diseases are diagnosed. In particular, the possibility that these methods will help us identify mineralocorticoid-induced hypertension is of interest to us.

Resistant hypertension is a state of excess MR activation in many instances, as judged by volume expansion^17^ and response to a MR antagonist.^18^ This MR antagonist responsiveness is not limited to patients with primary aldosteronism. More tailored treatment of resistant hypertension—and phenotyping of other forms of potentially MR-related hypertension^19^—will be facilitated by the further development of approaches such as ours.

A key barrier to progress in the field of urinary extracellular transcriptional biomarkers is the identification of a proper normalization strategy. In many cases, urinary creatinine is used for normalization purposes given its essentially constant rate of accumulation in urine. In this study, we observed no significant difference in urinary creatinine during low- and high-sodium diet. Therefore, differences in urinary concentration have been avoided, perhaps owing to study design; in essence, participants served as their own controls with regard to urinary concentration. This hypothesis is supported by the lack of a strong or consistent relationship between urinary creatinine and detected Ct values in our study population. However, it is possible that in random populations, normalization to creatinine will be useful. A study by Seo et al^20^ suggests that in some settings, even cross-sectional studies, urinary mRNA will be accurately quantifiable without normalization to another molecule.

Although proteins in urine supernatant have been extensively studied, the study of US-mRNA as a biomarker remains novel. Previous studies have characterized protein changes in urinary EVs under states of high and low aldosterone secretion or aldosterone infusion.^21–23^ These other methods measure MR-associated targets after protein translation. They do not directly measure MR activation per se, the immediate consequence of which is transcriptional activity. The method outlined in this article directly measures transcripts for genes known to be regulated by MR activation in animal models. Low-sodium dietary condition, higher serum aldosterone concentrations, and lower urinary sodium-creatinine ratio were each associated with lower C_t_ values of several MR target genes, indicating the presence of more copies of those targets in cell-depleted urine. These findings agree with our a priori expectation about the direction of change in MR-regulated gene expression after sodium loading. This work supports the use of US-mRNA to determine the physiological state of the kidney. US-mRNA may be useful for purposes of biomarker identification aimed at improving clinical precision in the diagnosis and treatment of diseases, including states of excess MR activation, such as many instances of resistant hypertension.

## Sources of Funding

Dr Byrd was supported by National Institutes of Health grant K23HL128909 and the Michigan MTRAC Kickstart Award.

## Disclosures

Drs Byrd, Rainey, and Auchus are inventors of a provisional patent related to detecting mineralocorticoid receptor activation. Dr Zocco is an employee of a company, Exosomics Siena SpA, that develops sample preps in the field of liquid biopsy. He is an inventor of patents owned by this company. The other authors report no conflicts.

## Supplementary Material
