# Supplementary material for: Human Urinary mRNA as a Biomarker of Cardiovascular Disease: A Proof-of-Principle Study of Sodium Loading in Prehypertension
Source: Circ Genom Precis Med. 2018 Sep 13;11(9):e002213. doi: 10.1161/CIRCGEN.118.002213 (PMC6760265; doi:10.1161/CIRCGEN.118.002213)

SUPPLEMENTAL MATERIAL

Supplemental Methods

Transcriptomic comparisons

Summarized expression data from human urinary extracellular vesicles and GTEx human kidney cortex, human brain, and human bladder samples were retrieved from the recount2[1] database and analyzed in R 3.4.3. Kidney-enriched and ubiquitous genes sets were taken from Uhlén et al.[2] Regularized log transformation was applied to raw counts with 'DESeq2' package,[3] and this measure was used for correlation analyses with Spearman's method ($P<0.005$ was considered significant). One-sided two-sample Kolmogorov-Smirnov test was applied between groups to calculate correlation indices ($P<0.001$ was considered significant).

Study participants

Eighteen otherwise healthy participants with pre-hypertension (120/80 ≤ BP < 140/90) were assigned to consume a low-sodium (20 mmol/day) diet, prepared in an on-site metabolic kitchen, for four days. Thirteen participants were male and five female, with an average age of 26.8 years. Blood and urine samples were collected while fasting on the fifth morning after initiation of the low-sodium diet. Once low-sodium samples were collected, patients were intravenously infused with sodium and provided with a high-sodium meal. Patients were sent home overnight with a urine collection container and an additional high sodium meal; overnight urine and a final fasting blood sample were collected the next morning. Laboratory evaluations performed on patient blood or urine samples included serum aldosterone concentration and plasma renin activity; serum sodium, potassium, and creatinine; urinary creatinine, and urinary sodium. Heart rate and blood pressure were measured using an ambulatory blood pressure monitor.

Isolation of RNA from urine supernatant

Frozen urine samples from the low-sodium and sodium-loaded conditions (approximately 4-5 mL) were retrieved from -80°C storage and thawed at room temperature. Once thawed, samples were vortexed vigorously for 30 seconds in order to break up uromodulin protein aggregates and resuspend extracellular vesicles into solution. Samples were then prepared for RNA isolation using an Exosome RNA Isolation Kit (Norgen Biotek, Thorold, Ontario). Per manufacturer's recommendations, samples were centrifuged at 1000 rpm (approximately 200 x*g*) for 10 minutes to avoid shearing cells. The supernatant was transferred to a fresh tube, and then centrifuged at 2500 rpm (approximately 1200 x*g*) for 10 minutes in order to pellet cells. The manufacturer's recommended protocol was then followed, with one modification: all centrifugation steps were performed at 4°C. Once isolation was complete, each 50 µL RNA sample was immediately stored at -80°C until used in qPCR experiments.

qPCR Assay Design

To achieve high sensitivity and specificity, we designed locked nucleic acid probe-based qPCR assays. cDNA sequences for MR target genes and control genes were retrieved from the Ensembl genome browser (http://www.ensembl.org/index.html). Each target gene sequence was archived in Seqbuilder (Lasergene, Madison WI) to facilitate annotation of primer and probe binding sites. We selected the locked nucleic acid probes from a universal probe library according to the target-gene cDNA sequences (http://qpcr.probefinder.com/organism.jsp; Roche, Basel, Switzerland). Once appropriate probes had been selected, the 200 bp region surrounding each probe was entered into Primer3 software, and appropriate primer pairs were designed to span an intron. We confirmed that the primers did not amplify DNA at 40 cycles of PCR (not shown).

Due to the presence of RNases in the urine, fragmentation of RNA was a concern. Bioanalyzer (Agilent Technologies) analysis revealed urinary RNA to comprise small fragments less than 200 bp. Therefore, we employed a strategy of probing multiple sites along the relevant gene sequences to increase the likelihood of detecting any truncated RNA molecules. We designed up to three separate primer sets -- when possible in view of all specificity-driven assay design constraints -- for each gene of interest, positioned to amplify distinct regions in these genes. We designed three sets of primer-probe assays for: *NR3C2*, *SCNN1A, SCNN1B, SCNN1G, SGK1, UMOD*. Genes for which we were able to design one or two primer-probe assays within the design constraints included: *AQP1, AQP2, GAPDH, HSD11B2, TSC22D3.*

To distinguish primer sets within the same target gene of interest, we used the following naming convention: gene name, followed by "-" and a number. For example, the three assays within the *SCNN1A* gene are named *SCNN1A*-1, *SCNN1A*-2, and *SCNN1A*-3.

Reverse Transcription and Pre-amplification of RNA

RNA samples were retrieved from -80°C storage and thawed on ice. While samples were thawing, reverse transcription master mix was prepared using the High-Capacity cDNA Reverse Transcription kit (Applied Biosystems); components were combined per manufacturer's instructions. 10 µL aliquots of master mix were then combined with 10 µL of RNA template for a final reaction volume of 20 µL. The reactions were run on a thermal cycler following the RT protocol outlined in Supplemental Figure 1.

The low nucleic acid content of urine prompted us to evaluate whether target-specific PCR amplification prior to qPCR was required in order to detect molecules by qPCR. In our preliminary experiments, pre-amplification of cDNA was found to improve reliability of target sequence detection. Therefore, for the study samples, pre-amplification reactions were set up

once RT was complete. Target-specific primer sequences were generated for each gene of interest (Integrated DNA Technologies, Coralville, IA), and 25 µL PCR reactions set up using the protocol outlined in the iTaq DNA Polymerase kit (BioRad); 6.25 µL of cDNA template was added to each reaction. Fifteen PCR cycles were performed following protocol outlined in Supplemental Figure 1.

Detection of Biomarker Candidates Using Fluorescence Reporter Probe qPCR

qPCR reactions were prepared using the same target-specific primer sequences used in the pre-amplification reactions. TaqMan 2x Universal PCR Master Mix was combined with primers and Universal Probe Library probes (Roche), and 15 µL aliquots were loaded onto 384 well qPCR plates. 5 µL of pre-amplified cDNA template was then added to each well. PCR reactions were carried out on a 7900HT thermal cycler (Applied Biosystems). Human kidney RNA (Clontech) and no-template controls were included on each plate as positive and negative controls, respectively. We assayed commercial human kidney RNA (Clontech) within each plate. The technical replicates were averaged within each plate and these results were used to adjust Ct values on each plate to account for any interplate differences.

Examination of using Urinary Creatinine for mRNA Normalization

The optimal normalization strategy for urine supernatant mRNA assays is currently an open question. Historically, normalization to urinary creatinine has been performed when measuring a wide variety of urinary analytes. Normalization to urinary creatinine attempts to measure analytes independent of any effect of varying urinary concentration. Thus, we considered the possibility that the C_t value of urinary supernatant mRNA molecules would vary according to the urinary creatinine. Urinary creatinine was similar during low-sodium diet and after sodium loading (Supplemental Figure 2). Moreover, no clear or consistent relationship was observed

between urinary creatinine and C_t value for the RNA molecules we assayed (Supplemental Figure 3). Moreover, relative quantitation has been found to be unnecessary even in a cross-sectional study of urinary mRNA.[4] For these reasons, C_t values are presented without normalization to another molecule.

Statistics

For the analysis of the clinical study, data are presented as means ± standard deviation. Between-group comparisons were made using the paired *t* test. Technical replicates' correlation with each other was analyzed using the Pearson correlation coefficient. C_t values from different qPCR assays within the same gene were evaluated using Pearson correlation coefficient, as was the relationship between C_t values and aldosterone or urinary sodium-creatinine ratio. A subset of assays probed participants' urine for the same gene target under the same dietary condition, but on a different qPCR plate (as an additional control)These repeated assays served as an additional internal control. To avoid counting these observations twice, all other analyses include only one plate's results for assays that were repeated on two plates for the same participant under the same dietary condition (as an internal control). The results retained were from the plate that either produced the most detected C_t values, or in the case of a tie, results from the plate prepared first were selected. We did not impute or otherwise assign a value when no mRNA was detected. A two-sided $P<0.05$ was considered significant, except where a more stringent threshold (arising from Bonferroni correction) is specified. Data were analyzed in R (R Foundation for Statistical Computing, Vienna, Austria).

Definition of Successful qPCR Result

We evaluated whether our technical replicates were tightly grouped, as expected if these assays performed well. We observed that when 2 or 3 of the technical triplicates for a sample yielded a

C_t value, the replicates were similar. For example, the median span of C_t values measured within sets of technical replicates was 0.14 cycles, with 75% of replicate sets spanning 0.23 cycles or less. Pearson correlation coefficients between replicates 1 & 2 and between replicates 2 & 3 were calculated for each assay. The correlation coefficients were greater than or equal to 0.990 in 86.7% of the 75 comparisons in this matrix (Supplemental Figure 7). Of the 858 technical triplicate sets constituting the results of our assays, there was one we excluded in subsequent analyses on the grounds of technical failure in view of results ranging from "not detected" to Ct values of 16.31 and 29.98 (one technical replicate set for *SCNN1A*-3, visible in Supplemental Figure 7). Of the remaining 857 technical triplicate sets, we accepted the 658 technical triplicates yielding three (98.0% of accepted values) or two (2.0% of accepted values) Ct values.

Distribution of C_t Values

Focusing on the 17 individuals from whom we could assay urinary aliquots by qPCR for both high- and low-sodium diet conditions, we examined the distribution of all C_t values passing quality control. In this analysis, we included target genes (MR target genes) & control genes (not expected to respond to MR activation; Supplemental Table 2) during low-sodium diet and after sodium loading. After calculating the mean of the technical replicates, the 658 C_t values passing our quality control filter tended to segregate into two bins: 1) between 20-30 cycles or 2) undetected, with few results falling below 20 or above 30 cycles. 95% percent of detected C_t values fell between 19.4 and 30.9, within a total range of 15.9 - 37.7 cycles. Although some correlations between age and specific gene products' C_t values were marginally statistically significant, we did not find compelling evidence of a relationship between age or sex and C_t values for the gene products we assayed.

Comparison of C_t Values Detected By Different Primers Within the Same Gene

When feasible according to our stringent primer-probe design principles, 3 assays were designed with a gene. We compared the results of different qPCR assays within each gene using Pearson correlation coefficient. As expected, we found high pairwise correlations between results of the different assays within a gene (Supplemental Figure 8).

References:

1. Collado-Torres L, et al. Reproducible RNA-seq analysis using recount2. *Nat Biotechnol*. 2017;35:319-321.

2. Uhlen M, et al. Transcriptomics resources of human tissues and organs. *Mol Syst Biol*. 2016;12:862.

3. Love MI, et al. Moderated estimation of fold change and dispersion for RNA-seq data with DESeq2. *Genome Biol*. 2014;15:550.

4. Seo JW, et al. Both absolute and relative quantification of urinary mRNA are useful for non-invasive diagnosis of acute kidney allograft rejection. *PLoS One*. 2017;12:e0180045.

Supplemental Table 1. Sample data accessions

Sample type	Accessions
Human urinary extracellular vesicles	SRR1185102
Human kidney cortex	SRR1325483, SRR1443092, SRR1362263, SRR1105272, SRR1469746, SRR1071807, SRR1490658, SRR1452888, SRR1089504, SRR1080366, SRR1340662, SRR1433066, SRR1328447, SRR1329154, SRR1468426, SRR1380931, SRR821356, SRR809943, SRR1445835, SRR1442708, SRR1420649, SRR810007, SRR1447631, SRR1500261, SRR1416516, SRR1432650, SRR1317086, SRR1396700
Human brain	SRR2166176, SRR2167642, SRR607445, SRR663753, SRR1485892, SRR1455060, SRR664854, SRR1337909, SRR1397094, SRR817686

Supplemental Table 2. List of primers used in this study and their associated Universal Probe Library Probe (Roche).

Primer	Sequence	UPL Probe #
***AQP1*-1 F**	gcctctctgtagcccttgg	79
***AQP1*-1 R**	aaggaccgagcagggttaat	
***AQP1*-2 F**	cctccctgactgggaactc	81
***AQP1*-2 R**	ggagggtcccgatgatct	
***AQP2*-1 F**	ctgctctctccataggcttctc	81
***AQP2*-1 R**	tttgccagtgacgacagc	
***GAPDH*-1 F**	ccccggtttctataaattgagc	68
***GAPDH*-1 R**	caccttccccatggtgtct	
***HSD11B2*-1 F**	ggtcaaggtcagcatcatcc	71
***HSD11B2*-1 R**	agcagctcttgaggcaggt	
***NR3C2*-1 F**	ttttcttcaaaagagcagtgga	11
***NR3C2*-1 R**	ggacaattctttcgtcgaatct	
***NR3C2*-2 F**	tgaccaagctgctggactc	62
***NR3C2*-2 R**	actctaccttcagcgcatgg	
***NR3C2*-3 F**	gtgggcaaaggtacttccag	78
***NR3C2*-3 R**	tggctgttcgtatgtttgtacg	
***SCNN1A*-1 F**	tctgcaccctcaatccctac	25
***SCNN1A*-1 R**	caggtcaaagagcgtctgct	
***SCNN1A*-2 F**	caaccaggtctcctgcaac	31
***SCNN1A*-2 R**	gaaagtatagcagtttccatacatcg	
***SCNN1A*-3 F**	ggcagtgatgttcctgttga	62
***SCNN1A*-3 R**	tcctggaagcaggagtgaata	
***SCNN1B*-1 F**	caccaatatcaccctgagca	66
***SCNN1B*-1 R**	agattcgagagcagccagac	
***SCNN1B*-2 F**	gccaaccctggaactgaat	72
***SCNN1B*-2 R**	gacctctgctcgtgaagcat	
***SCNN1B*-3 F**	caatgctagccccttcaagt	85
***SCNN1B*-3 R**	caggacagcttccatcagc	
***SCNN1G*-1 F**	gagtgacgtgccaatcagg	50
***SCNN1G*-1 R**	gtctggaagcatgaatgaagg	
***SCNN1G*-2 F**	gggatgctagcccgagag	68
***SCNN1G*-2 R**	cgaggatgggactttgagg	
***SCNN1G*-3 F**	tgcttctttgatggagtgtcc	72
***SCNN1G*-3 R**	ggtgctgagaatggtctcattt	
***SGK1*-1 F**	tttccaaagaggggttctcc	2
***SGK1*-1 R**	tggcatgattacatggctct	
***SGK1*-2 F**	tcaggagcctgagcttatgaa	22
***SGK1*-2 R**	ttagcatgaggattggacga	
***SGK1*-3 F**	gacaggactgtggactggtg	24
***SGK1*-3 R**	tttcagctgtgtttcggcta	
***TSC22D3*-1 F**	ccatcctgctcttcttccac	36
***TSC22D3*-1 R**	atggcctgttcgatcttgtt	
***TSC22D3*-2 F**	gatcgaacaggccatggat	68
***TSC22D3*-2 R**	tcttctccaccagctctcg	
***UMOD*-1 F**	gccagtgcaaacaggacttc	1
***UMOD*-1 R**	gtcattggccccacattc	
***UMOD*-2 F**	aagtacttcatcatccaggacaga	22
***UMOD*-2 R**	aacatctggacggaaaatcg	
***UMOD*-3 F**	cactgacacctcagaagcaaga	88
***UMOD*-3 R**	tgacaggtgcacgtcgtaac	

Supplemental Table 3. List of 55 kidney-enriched genes used in the RNA-Seq subset analysis.

ABCA4	*CYS1*	*NAT8*	*SLC17A1*	*SLC6A13*
AQP2	*DMRT2*	*NPHS2*	*SLC22A11*	*SLC6A18*
AQP6	*DUSP9*	*OR2T10*	*SLC22A12*	*SPP1*
ATP6V0D2	*EMX1*	*PAX2*	*SLC22A13*	*TMEM174*
ATP6V1G3	*FOXI1*	*PDZK1*	*SLC22A2*	*TMEM207*
BSND	*FXYD2*	*PDZK1IP1*	*SLC22A6*	*TMEM213*
CDH16	*FXYD4*	*PTH1R*	*SLC22A8*	*TMEM27*
CLCNKB	*HMX2*	*REN*	*SLC34A1*	*TMEM52B*
CLDN16	*KCNJ1*	*SLC12A1*	*SLC47A2*	*TRPV5*
CRYAA	*MCCD1*	*SLC12A3*	*SLC4A9*	*UGT1A9*
CYP27B1	*MIOX*	*SLC13A3*	*SLC5A10*	*UMOD*

Supplemental Table 4. Clinical characteristics of study participants during low- and high-sodium diet.

	Measurement	Low Sodium Diet, Mean±SD (n)	Sodium Loaded, Mean±SD (n)	*P* value
**Serum Assays**				
	Sodium, mmol/L	137.2±2.0 (18)	140.4±1.6 (18)	<0.001
	Potassium, mmol/L	4.2±0.3 (18)	4.3±0.4 (18)	0.09
	Creatinine, mg/dL	1.0±0.1 (18)	0.9±0.1 (18)	<0.001
**Plasma Assays**				
	Plasma renin activity, ng/(mL*hour)	4.1±2.0 (18)	0.7±0.6 (16)	<0.001
	Serum aldosterone, ng/dL	32.2±14.8 (18)	11.2±4.8 (17)	<0.001
	Aldosterone-renin ratio	14.1±21.4 (18)	23.6±14.1 (15)	0.19
**Urinary Assays**				
	Sodium, spot, mmol/L	15.2±9.2 (17)	85.7±38.5 (18)	<0.001
	Creatinine, mg	102.3±64.2 (18)	99.7±59.6 (18)	0.82
	Sodium-creatinine ratio	0.2±0.1 (17)	1.1±0.7 (18)	<0.001
**Hemodynamic Parameters**				
	Heart rate, beats per minute	77.5±9.1 (17)	76.8±8.5 (17)	0.63
	Systolic blood pressure, mmHg	122.1±5.4 (17)	122.2±9.2 (17)	0.93
	Mean arterial pressure, mmHg	90.5±5.1 (17)	86.8±6.3 (17)	0.03
	Diastolic blood pressure, mmHg	73.6±7.0 (17)	69.3±6.7 (17)	0.01

Supplemental Figure 1. Illustration of the procedure used in extracting urine superatant mRNA and performing qPCR assays.

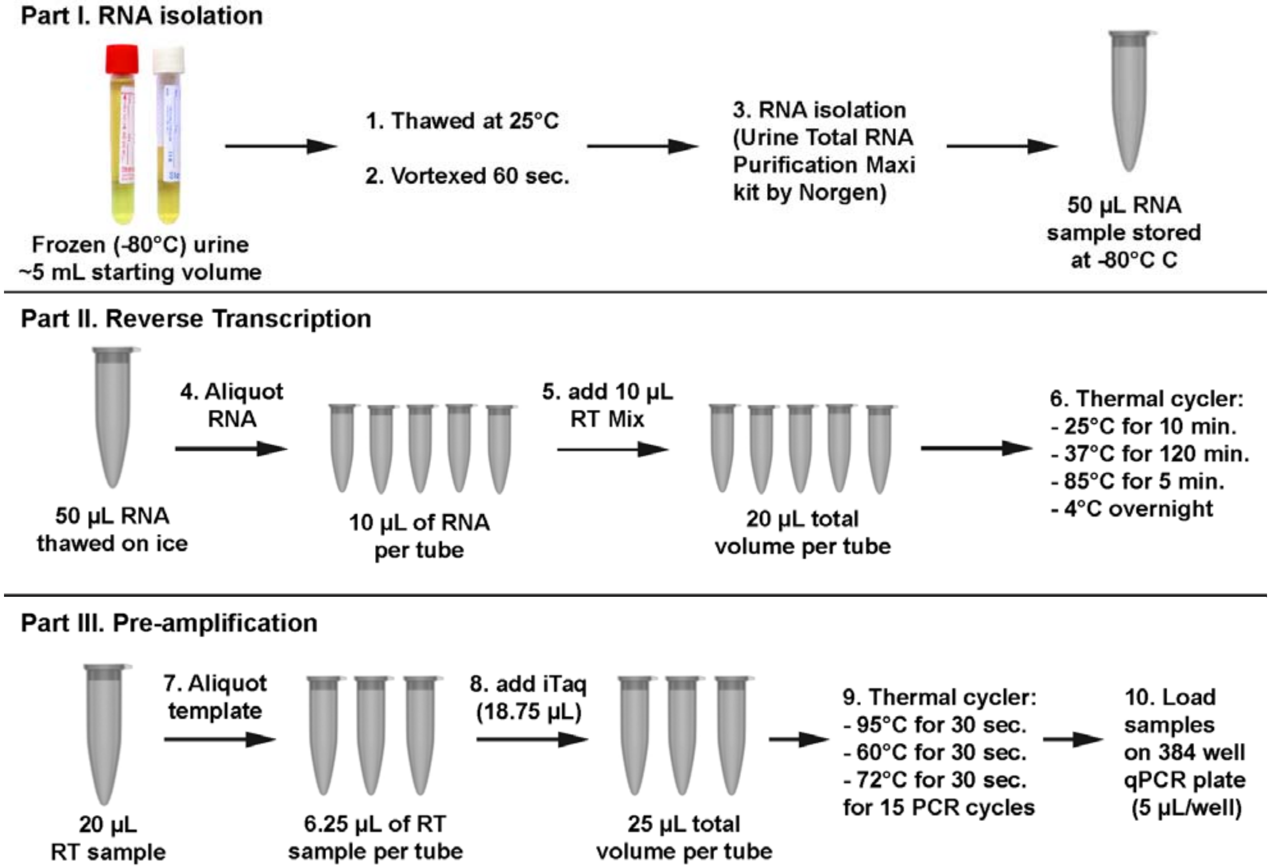

Supplemental Figure 2. Urinary creatinine in low-sodium and sodium-loaded phases of the study.

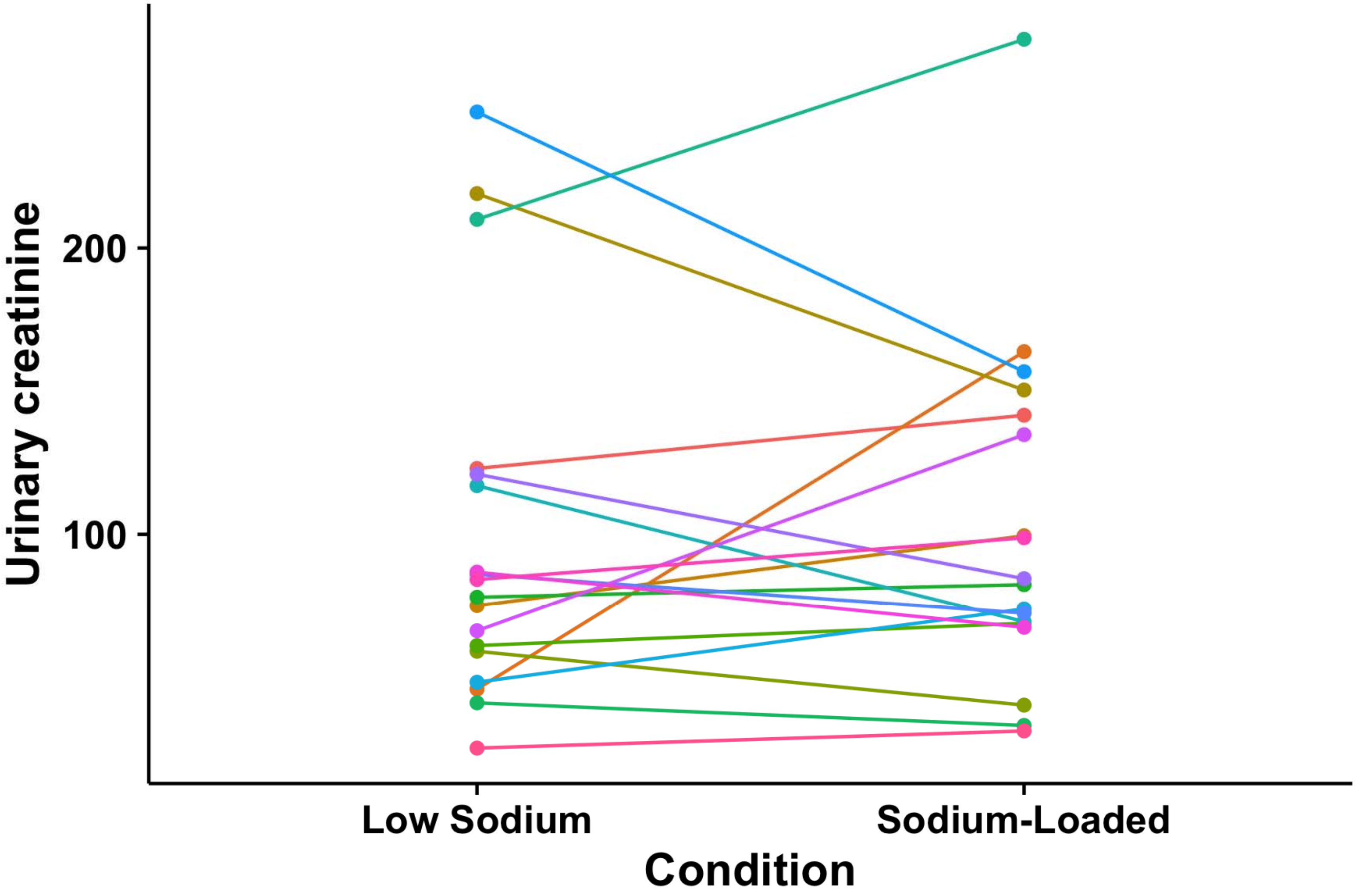

Supplemental Figure 3. **Relationship between log-transformed urinary creatinine and C_t value for the genes assayed.**

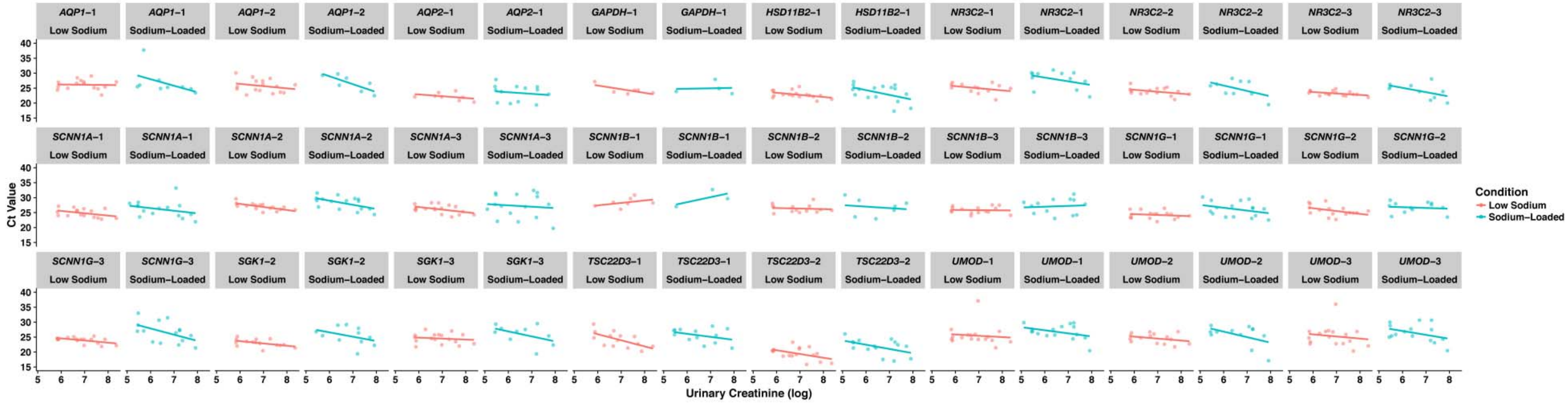

Supplemental Figure 4. Log urinary sodium-urinary creatinine ratio, stratified by sodium condition (low sodium compared to sodium-loaded condition).

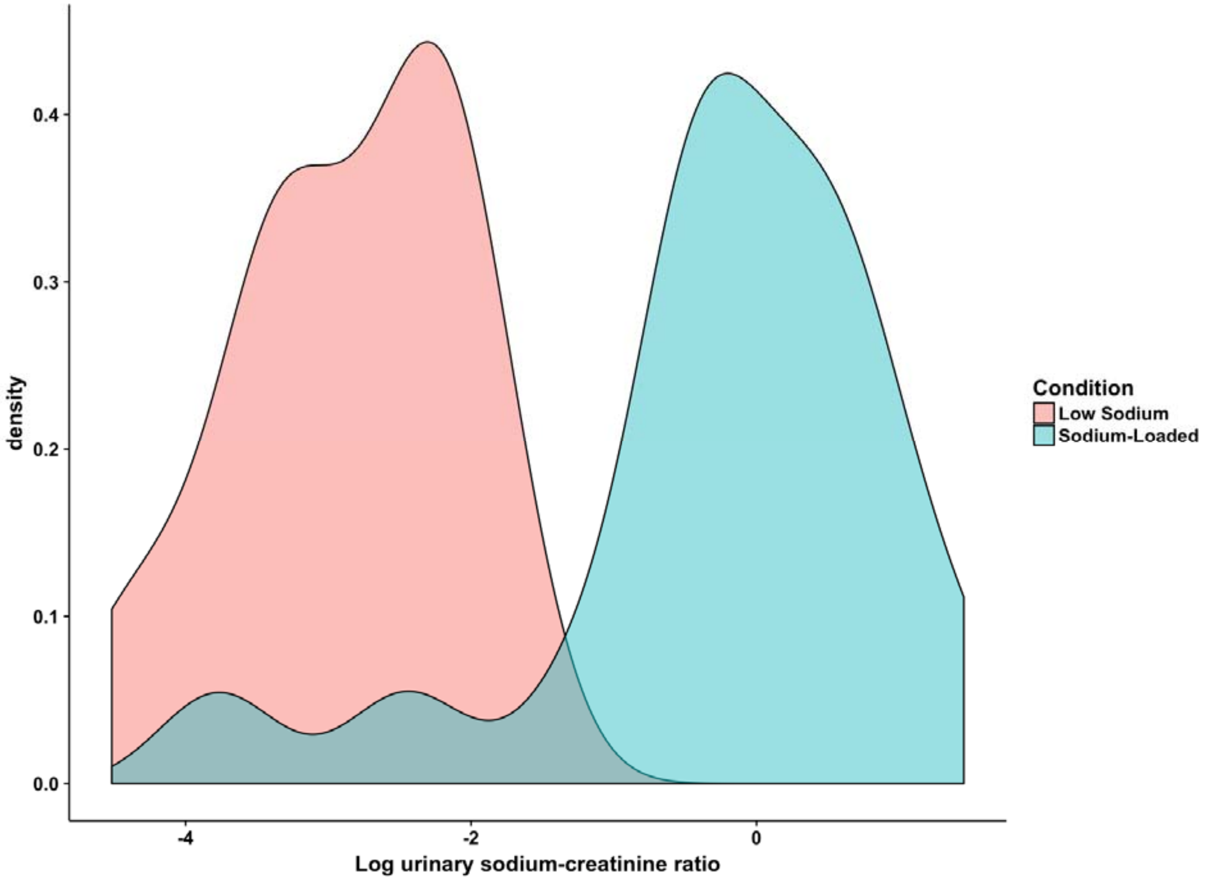

Supplemental Figure 5. Number of replicates within technical triplicate sets yielding a C_t value. Data are shown by qPCR assay.

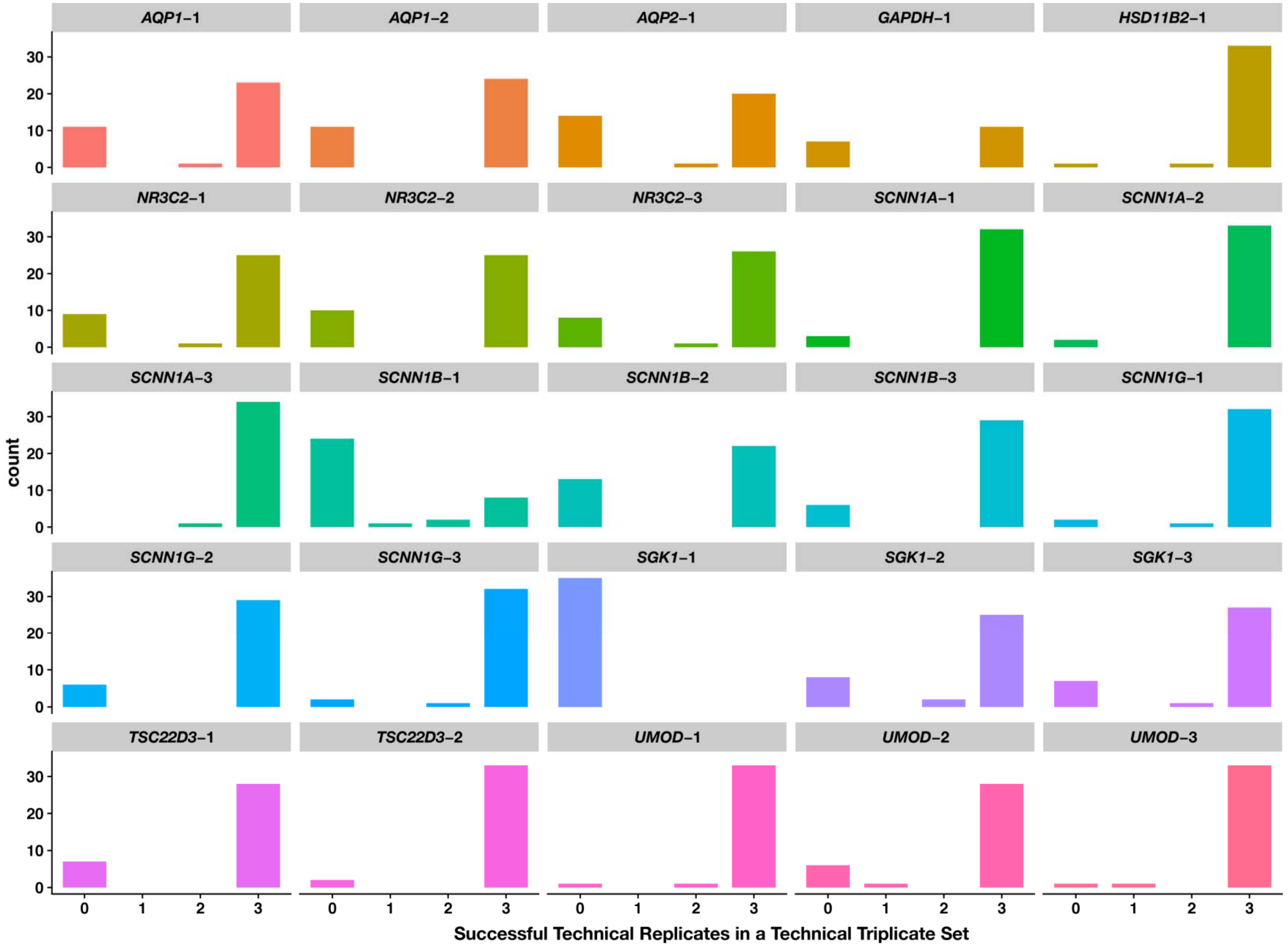

Supplemental Figure 6. Normal Q-Q plot of C_t values aggregated across 25 qPCR assays, 17 study participants, and both sodium conditions (low sodium and sodium-loaded), revealing a generally normal distribution with few outliers at extreme C_t values.

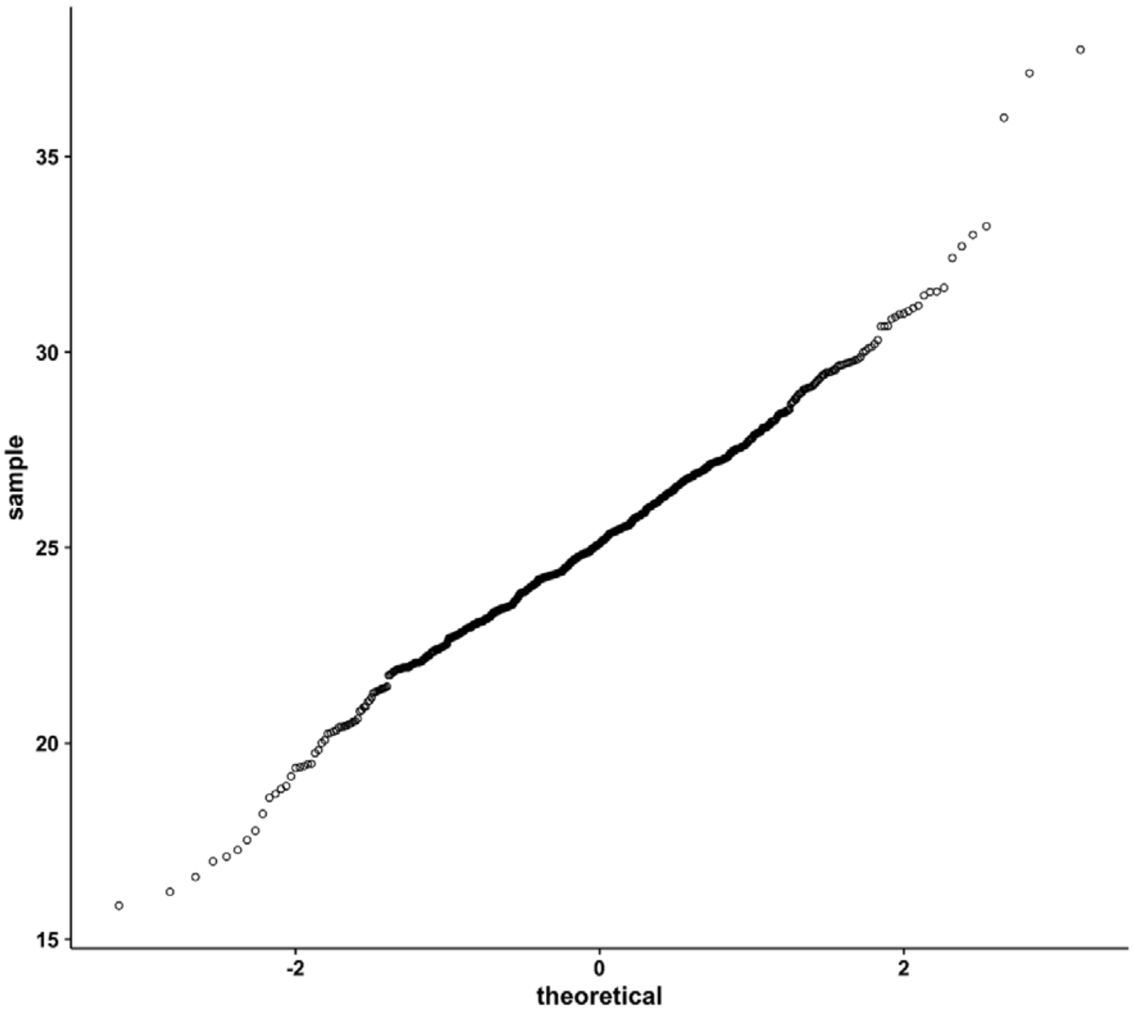

Supplemental Figure 7. Comparison of 1st, 2nd, and 3rd C_t values comprising technical triplicates of urinary supernatant mRNA qPCR assays. Numbers represent Pearson correlation coefficient for comparisons within each triplicate.

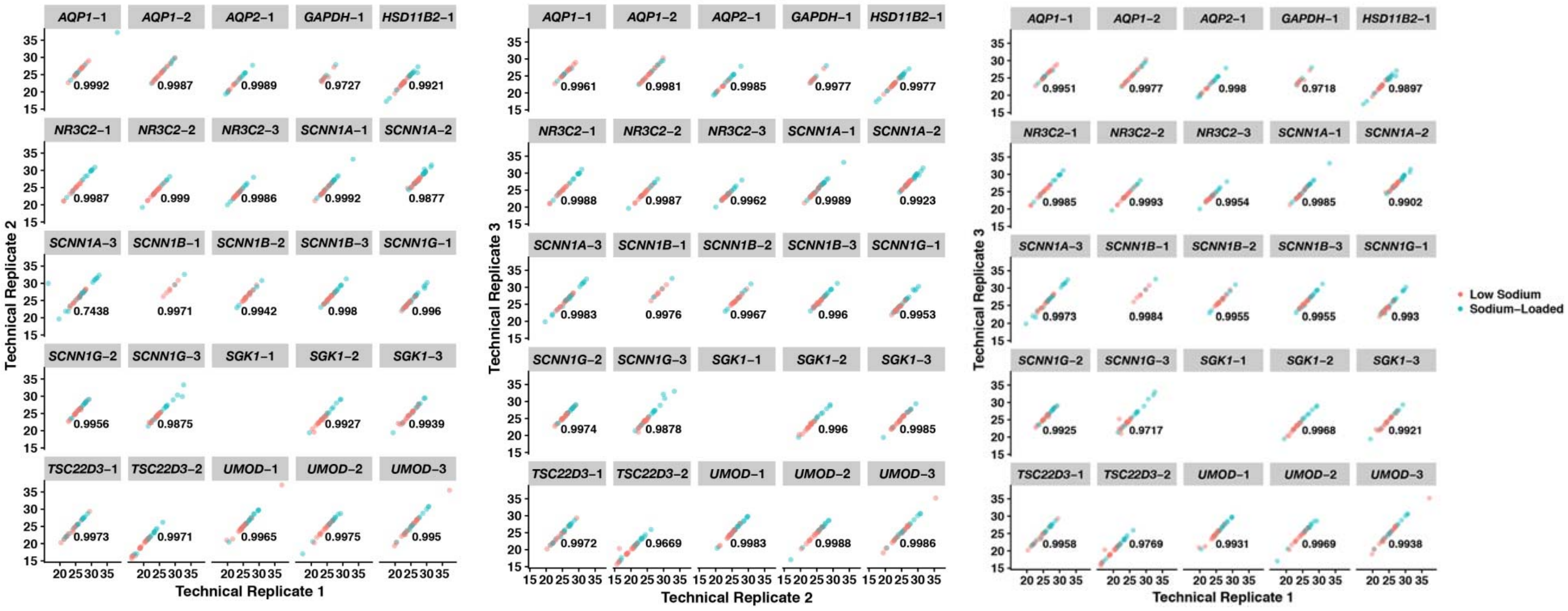

Supplemental Figure 8. Pearson correlation coefficient between different qPCR assays within the same gene.

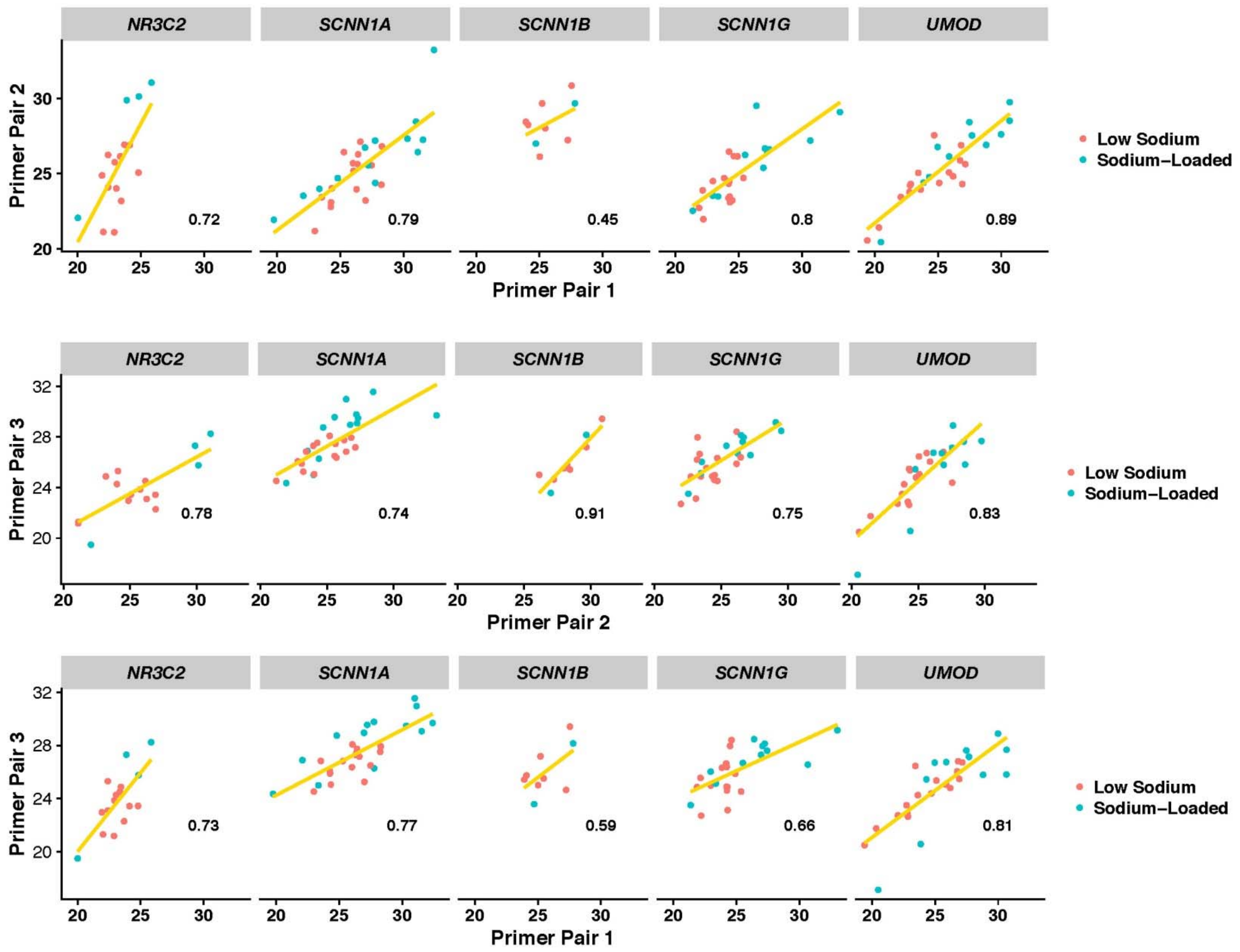

Supplement: Supplementary file 1 [file hcg-11-e002213-s001.pdf]
